# Spontaneous physical functional recovery after hospitalization for COVID-19: insights from a 1 month follow-up and a model to predict poor trajectory

**DOI:** 10.3389/fmed.2023.1212678

**Published:** 2023-07-20

**Authors:** Oleksii Honchar, Tetyana Ashcheulova

**Affiliations:** Department of Propedeutics of Internal Medicine No. 1, Fundamentals of Bioethics and Biosafety, Kharkiv National Medical University, Kharkiv, Ukraine

**Keywords:** COVID-19, hospitalization, convalescence, physical functional performance, recovery of function, walk test, clinical decision rules, machine learning

## Abstract

**Background:**

Long COVID syndrome has emerged as a new global healthcare challenge, with impaired physical performance being a prominent debilitating factor. Cardiopulmonary rehabilitation is a mainstay of management of symptomatic post-COVID patients, and optimization of candidate selection might allow for more effective use of available resources.

**Methods:**

In order to study the natural dynamics and to identify predictors of physical functional recovery following hospitalization for COVID-19, 6 min walk test was performed pre-discharge in 176 patients (40% hypertensive, 53% female, mean age 53.2 ± 13.5 years) with re-evaluation at 1 month.

**Results:**

Six min walk distance and the reached percent of predicted distance (6MWD%) were suboptimal at both visits—396 ± 71 m (68.7 ± 12.4%) pre-discharge and 466 ± 65 m (81.8 ± 13.6%) at 1 month. Associated changes included significant oxygen desaturation (2.9 ± 2.5 and 2.3 ± 2.2%, respectively) and insufficient increment of heart rate during the test (24.9 ± 17.5 and 28.2 ± 12.0 bpm) that resulted in low reached percent of individual maximum heart rate (61.1 ± 8.1 and 64.3 ± 8.2%). Automatic clusterization of the study cohort by the 6MWD% changes has allowed to identify the subgroup of patients with poor “low base—low increment” trajectory of spontaneous post-discharge recovery that were characterized by younger age (38.2 ± 11.0 vs. 54.9 ± 12.1, *p* < 0.001) but more extensive pulmonary involvement by CT (43.7 ± 8.8 vs. 29.6 ± 19.4%, *p* = 0.029) and higher peak ESR values (36.5 ± 9.7 vs. 25.6 ± 12.8, *p* < 0.001). Predictors of poor recovery in multivariate logistic regression analysis included age, peak ESR, eGFR, percentage of pulmonary involvement by CT, need for in-hospital oxygen supplementation, SpO_2_ and mMRC dyspnea score pre-discharge, and history of hypertension.

**Conclusion:**

COVID-19 survivors were characterized by decreased physical performance pre-discharge as assessed by the 6 min walk test and did not completely restore their functional status after 1 month of spontaneous recovery, with signs of altered blood oxygenation and dysautonomia contributing to the observed changes. Patients with poor “low base—low increment” trajectory of post-discharge recovery were characterized by younger age but more extensive pulmonary involvement and higher peak ESR values. Poor post-discharge recovery in the study cohort was predictable by the means of machine learning-based classification model that used age, history of hypertension, need for oxygen supplementation, and ESR as inputs.

## 1. Introduction

COVID-19 pandemic continues to present a protracted challenge to the healthcare systems and economies worldwide. Despite a significant reduction in mortality as the result of global vaccination campaigns and natural evolution of prevailing SARS-CoV-2 types, concerns are being increasingly raised in relation to the high incidence of long-lasting symptoms among COVID-19 convalescents (1, 2). This scenario has been attributed several generic names (3–5), with “long COVID” being most frequently used. According to different sources, 5% to 48% patients continue to report at least one symptom persisting beyond 3 months after disease onset (2, 6), with the rate getting as high as 76% among those who required hospitalization and up to 81% after intensive care unit admission (7). An important aspect of this problem is also related to potentially debilitating effect of some of the typical long COVID sequelae that include dyspnea, fatigue, and signs of cognitive dysfunction (1, 2, 8).

Assessment of the origin of physically limiting symptoms in the post-acute COVID-19 setting is frequently complicated due to significant heterogeneity of possible underlying mechanisms. The latter might include signs of pulmonary restriction and decreased diffusing capacity (9, 10), cardiac dysfunction (2, 11), and a significant share of unexplained cases, the rate of which is generally higher in non-severe COVID-19 convalescents (12).

As one of the consequences, to date no specific treatment has demonstrated beneficial effects for the prevention and treatment of long COVID-related dyspnea and fatigue (8, 13). The mainstay of post-acute management in these patients is presented by cardiopulmonary rehabilitation that has been shown to have positive effect on the dynamics of symptoms and functional status (14–16). At the same time, the number of convalescents typically exceeds the available programs capacity, and the real-life post-acute COVID-19 care would benefit from the possibility to identify the optimal candidates to enter supervised rehabilitation.

Another consequence of the described heterogeneity of long COVID physiology is the limited usability of parameters that specifically assess pulmonary or cardiac function for the monitoring of rehabilitation efficacy in the general cohort of post-acute patients. With availability, versatility, and extensive validation in a wide set of conditions being other important considerations, general physical performance assessment in the 6 min walk test (6MWT) (17–19) is a viable choice in this setting.

Thus, the objective of this study was to investigate the dynamics of spontaneous physical functional recovery as assessed by the 6MWT during 1 month follow-up after hospitalization for COVID-19 and to identify the pre-discharge predictors of poor improvement.

## 2. Materials and methods

### 2.1. Study design and population

**Figure 1:**
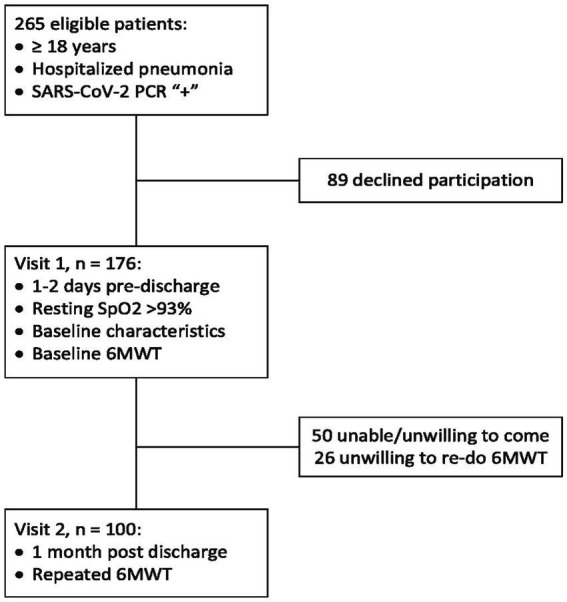
Study flow chart.

By design, this is a combined cross-sectional and longitudinal prospective observational single-center study that was carried out at Kharkiv City Hospital #13 (which is a regional pulmonological and COVID-19 treatment center that was serving the area of about 2.4 million people at the period of recruiting between January and November 2021). Eligibility criteria included the age of ≥18 years and hospitalization for pneumonia, with SARS-CoV-2 etiology being confirmed with a positive polymerase chain reaction test. Exclusion criteria are presented in the Supplementary material, and the study flow chart is shown in Figure 1. Of the 265 consecutive eligible patients who were invited to participate in the study, 176 were enrolled, and 100 patients have completed the study, presenting the final cohort.

The study was conducted in compliance with the standards of Helsinki Declaration and was approved by the ethical committee of Kharkiv National Medical University. All participants provided written informed consent.

### 2.2. Clinical data collection

The first visit was typically performed 1–2 days prior to discharge, in clinically stable and epidemically safe patients (resting capillary blood oxygen saturation (SpO_2_) >93% on room air, normal body temperature and absence of acute respiratory disease symptoms for ≥3 days starting from the 10th day after the symptoms onset) (20). During this visit, medical records were used to retrieve baseline demographic characteristics (age, gender), data on treatment, laboratory parameters, and computed tomography findings (percentage of pulmonary involvement was assessed using the methodology for the simplified RALE score as proposed by Wong et al. (21), mean value of the reported range was taken for analysis), and interview was performed to collect data on symptoms and medical history, followed by anthropometrical measurements and 6MWT. A follow-up visit for re-assessment of the severity of symptoms, changes in clinical parameters, and repeated 6MWT was carried out after 1 month of spontaneous recovery.

### 2.3. Six min walk test

6MWT was performed by a physician using a 20 m hallway and the standard methodology as recommended by the American Thoracic Society guidelines (22). The test was self-paced, with standard instructions provided before the start and every minute thereafter; no warm-up period, practice test or additional encouragement was used. SpO_2_ and peripheral pulse were constantly monitored and registered at the baseline and every 30 s throughout the distance via Bluetooth-connected pulse oximeter. Blood pressure was measured at the baseline, dyspnea and fatigue levels were assessed using modified Borg scale pre- and post-test. Classic sex-specific equations by Enright et al. (23) were used to calculate the individual predicted 6MWD.

The parameters taken for further analysis included the distance walked (6MWD), reached percent of individually predicted distance (6MWD%), increase of the absolute (Δ6MWD) and reached percent of predicted distance (Δ6MWD%) between visits, baseline (HR_base_) and maximal heart rate during the test (HR_max_), highest reached percent of the individual maximum heart rate that was calculated as HR_max %_ = 100% × HR_max_/(208–0.8 × age) (24) and the same parameter registered at the end of the test (HR_fin %_), baseline (SpO_2 base_), minimal (SpO_2 min_) and final (SpO_2 fin_) capillary blood oxygen saturation levels, and peak oxygen desaturation that was calculated as SpO_2 drop_ = SpO_2 min_ – SpO_2 base_.

### 2.4. Statistical analysis

The collected data was analyzed using StatSoft STATISTICA Version 12 statistical analysis software package. Data distribution was assessed using Shapiro–Wilk test. For all variables, descriptive statistics are reported as mean ± standard deviation (SD) or median (interquartile range) for normally distributed and skewed continuous variables, respectively. Categorical variables are reported as counts (percentages). Cross-sectional comparisons of continuous variables were made using independent samples t-test for normally distributed parameters and Mann–Whitney *U*-test for skewed variables; chi-square test was used to compare binary and categorical variables. Longitudinal comparisons were made using paired samples *t*-test or Wilcoxon signed-rank test. *p*-values reported were calculated using two-sided Fisher’s exact test, the differences were considered significant if *p* < 0.05. Cluster analysis was performed using *k*-means algorithm with 10-fold cross-validation. Logistic regression analysis was performed using a forward stepwise method; Somers’ D, Kolmogorov–Smirnov statistic, and receiver-operator characteristic (ROC) analysis were used to assess the quality of regression models, and Wald *p*-value to assess significance of included predictors.

Unsupervised machine learning (ML) approach was used to create classification models based on simple artificial neural networks (SANN). For each set of input variables, 500 predictive models were trained using the automated neural architecture search strategy and Broyden–Fletcher–Goldfarb–Shanno optimization algorithm with subsequent automatic ranging of the obtained models by their predictive performance. The latter was assessed as percentage of correctly classified cases from the training, test and validation subsets that were obtained by random sampling of the study cohort in the 70:15:15 proportion. For the final predictive model, 10-fold cross-validation was used, and ROC analysis performed. Sample size adequacy was evaluated post-hoc using combined approach that included assessment of model accuracy and the dataset effect size using Cohen’s *d* statistic (25).

## 3. Results

### 3.1. Baseline characteristics

The initial study cohort included 83 male and 93 female patients at the age of 53.2 ± 13.5 years, with hypertension and obesity being the most frequent comorbidities, followed by type 2 diabetes mellitus. Visit 1 was carried out at the median of 22 [18–27] days, and visit 2 at 52 [49–60] days after the symptoms onset. The patients who had completed visit 2 (*n* = 100) were representative of the general study population with the exception of slightly higher eGFR values—see Table 1 for detailed clinical characteristics.

**Table 1:** Demographics and clinical characteristics of the study participants.

Parameters	General cohort	Final cohort
Subjects	176	100
Female sex	93 (53)	44 (44)
Age, years	53.2 ± 13.5	50.9 ± 14.1
Height, cm	169.8 ± 9.1	171.8 ± 9.6
Weight, kg	84.5 ± 18.5	86.1 ± 16.0
BMI, kg/m^2^	29.1 ± 5.2	29.1 ± 4.6
*Comorbidities*		
Hypertension	70 (40)	41 (41)
Obesity	67 (38)	42 (42)
Diabetes mellitus, type 2	17 (10)	14 (14)
History of peptic ulcer	13 (7)	7 (7)
History of cancer	10 (6)	4 (4)
History of stroke/TIA	6 (3)	0 (0)
Chronic kidney disease	5 (3)	2 (2)
Chronic obstructive pulmonary disease	5 (3)	3 (3)
Bronchial asthma	4 (2)	2 (2)
Pulmonary emphysema	3 (2)	2 (2)
Angina pectoris	3 (2)	0 (0)
Chronic liver disease	2 (1)	1 (1)
Charlson comorbidity index	0.5 ± 0.8	0.5 ± 0.9
Active smoking status	29 (16.5)	24 (24)
Positive vaccination status (single dose)	7 (4)	4 (4)
Intensive care unit admission	9 (5.1)	6 (6)
Pulmonary tissue involvement by CT, %	32.5 ± 20.2	31.7 ± 18.6
Minimal SpO_2_ during hospitalization, %	89 [85–94]	88.4 ± 7.9
*Oxygen supplementation*		
Via nasal cannula	101 (57.4)	57 (57)
Noninvasive/invasive ventilation	9 (5.1)	6 (6)
*Laboratory parameters*		
Peak IL-6, pg/mL	10.0 [3.1–25.2]	11.7 [3.2–47.4]
Peak CRP, mg/L	24.0 [7.3–55.0]	26.0 [6.0–60.0]
Peak ESR, mm/h	30.6 ± 13.7	29.3 ± 12.8
Peak procalcitonin, ng/mL	0.06 [0.04–0.12]	0.06 [0.05–0.12]
Peak D-dimer, ng/mL	278 [154–508]	219 [156–414]
Peak creatinine, μmol/L	103.3 ± 22.3	99.7 ± 17.3
Lowest eGFR, mL/min/1.73m^2^	65.3 ± 18.6	72.7 ± 19.0
*Treatment*		
Remdesivir	82 (46.6)	43 (43)
Dexamethasone	155 (88.1)	84 (84)
Methylprednisolone pulse therapy	115 (65.3)	61 (61)
Antibiotics	152 (86)	84 (84)

*p* = 0.002 for eGFR, *p* > 0.05 for all other parameters. BMI, body mass index; TIA, transient ischemic attack; CT, computed tomography; SpO_2_, peripheral capillary oxygen saturation; IL-6, interleukin 6; CRP, C-reactive protein; ESR, erythrocyte sedimentation rate; eGFR, estimated glomerular filtration rate by CKD-EPI equation.

### 3.2. Physical performance in the 1 month follow-up

**Figure 2:**
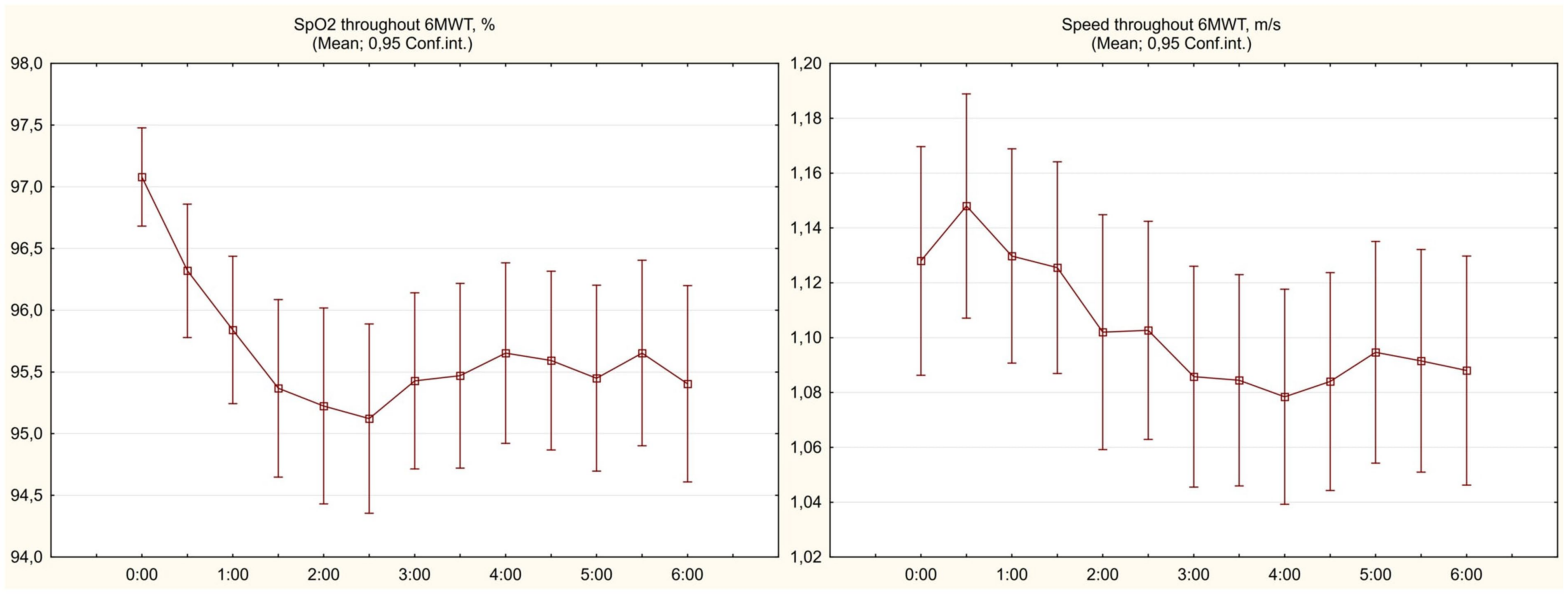
Capillary blood oxygen saturation and walking speed dynamics in 6 min walk test.

Baseline 6 min walk test has shown signs of significantly impaired functional status in observed COVID-19 survivors, with mean 6MWD% values of 68.7 ± 12.4%. All participants have completed the test; SpO_2_ levels remained >90% but typically showed an initial dip reaching the lowest values at 2:30 min with subsequent partial resolving on the background of adjustment of initial pace (see Figure 2). Table 2 summarizes the features of baseline 6MWT and provides comparison to the results of repeated test performed at 1 month.

**Table 2:** Dynamics of 6MWT parameters in COVID-19 survivors throughout a 1 month post-discharge follow-up.

Parameters	Visit 1 (pre-discharge)	Visit 2 (at 1 month)	Difference (95% CI)	2-sided *p*
*Baseline*				
Weight, kg	86.1 ± 16.0	88.6 ± 16.9	2.5 (1.8–3.1)	<0.001
BMI, kg/m^2^	29.1 ± 4.6	30.0 ± 4.9	0.8 (0.6–1.1)	<0.001
6MWD predicted	586 ± 106	581 ± 107	5 (4–6)	<0.001
SBP, mm Hg	133.7 ± 16.3	133.6 ± 15.5	−0.1 (−3.2 to 3.1)	0.970
DBP, mmHg	82.8 ± 11.5	81.8 ± 11.4	−1.0 (−2.9 to 0.8)	0.280
Heart rate, min^−1^	82.5 ± 12.7	80.9 ± 14.5	−1.6 (−4.7 to 1.6)	0.328
SpO_2 base_, %	97.1 ± 2.0	98.1 ± 0.8	1.0 (0.6–1.4)	<0.001
Baseline dyspnea^a^	1.5 ± 1.6	0.8 ± 1.1	−0.7 (−1.0 to −0.3)	<0.001
Baseline fatigue^a^	2.3 ± 2.1	1.3 ± 1.4	−1.0 (−1.4 to −0.5)	<0.001
*Throughout the test*				
6MWD, m	396 ± 71	466 ± 65	70 (61–78)	<0.001
6MWD%	68.7 ± 12.4	81.8 ± 13.6	13.1 (11.5–14.7)	<0.001
HR_max, min_^−1^	105.6 ± 14.7	110.5 ± 14.2	4.9 (1.9–7.9)	0.001
HR_max_ %	61.1 ± 8.1	64.3 ± 8.2	3.1 (1.4–4.8)	<0.001
HR increment, min^−1^	24.9 ± 17.5	28.2 ± 12.0	3.4 (0.3–7.0)	0.069
SpO_2 min_, %	94.2 ± 4.1	95.8 ± 2.4	1.6 (0.9–2.3)	<0.001
SpO_2 fin_, %	95.5 ± 3.8	97.2 ± 1.4	1.6 (1.0–2.3)	<0.001
SpO_2 drop_, %	2.9 ± 2.5	2.3 ± 2.2	−0.6 (−1.1 to −0.1)	0.022
Dyspnea at 6 min^a^	3.5 ± 2.1	3.0 ± 1.9	−0.6 (−1.2 to 0.0)	0.057
Fatigue at 6 min^a^	3.4 ± 1.9	2.2 ± 1.9	−1.1 (−1.9 to −0.4)	0.002

CI, confidence interval; BMI, body mass index; 6MWD, 6 min walk distance; SBP, systolic blood pressure; DBP, diastolic blood pressure; SpO_2 base_, baseline oxygen saturation; 6MWD%, reached % of predicted 6 min walk distance; HR_max_, maximal reached heart rate; HR_max_ %, reached percent of the individual maximum heart rate; SpO_2 min_, minimal oxygen saturation; SpO_2 fin_, oxygen saturation at 6 min; SpO_2 drop_, peak oxygen desaturation.

a As assessed by Borg scale.

6MWT parameters at 1 month were characterized by the universal improvement vs. pre-discharge visit, with 70 ± 43 m gain in absolute and 13.1 ± 7.9% in the reached percent of predicted 6MWD, steadily higher SpO_2_ values at the baseline and throughout the test, and lower subjective levels of fatigue and dyspnea. At the same time, both 6MWD% and HR_max_ % were remaining suboptimal showing modest mean values of 81.8 ± 13.6% and 64.3 ± 8.2, with insufficient heart rate increment throughout the test demonstrating no significant improvement between visits.

**Figure 3:**
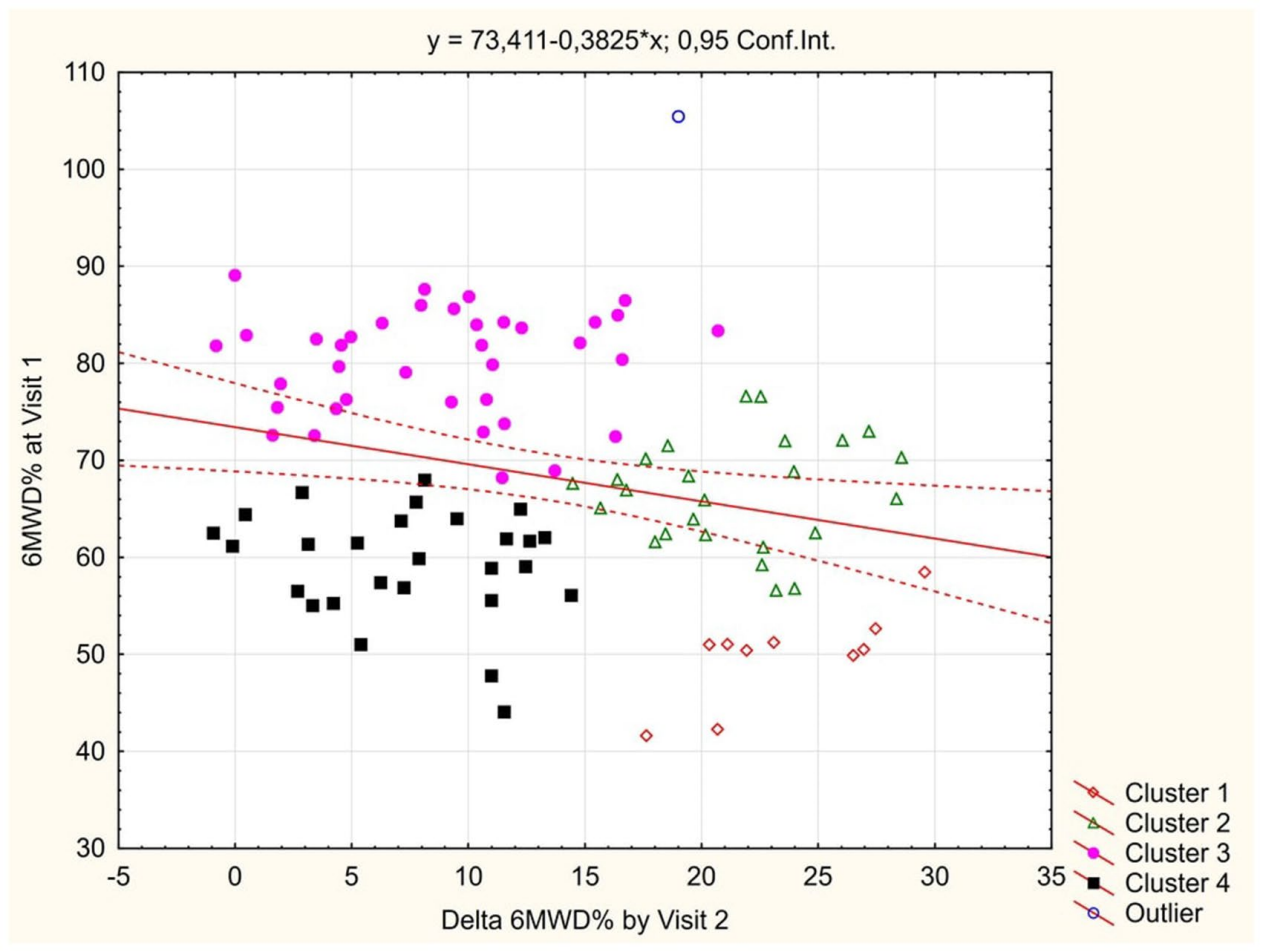
Clusterization of study participants by 6MWD% changes.

In order to identify study participants who had shown insufficient functional improvement during 1 month follow-up, we performed clusterization of patients by the values of baseline 6MWD% and Δ6MWD% between visits. The scatterplot demonstrating the results is shown at Figure 3.

Among the resulting four non-intersecting clusters of observed COVID-19 convalescents (training error = 0.19), cluster 1 (*n* = 10) was presented by patients who demonstrated the largest gain in 6MWD% between visits despite initially low values; cluster 2 (*n* = 25) was showing average baseline 6MWD% values but a high gain between visits; cluster 3 (*n* = 37) patients were showing lower improvement between visits that was explained by a high base effect; and cluster 4 (*n* = 27) participants were exhibiting the least favorable profile, where initially lower 6MWD% values were not compensated by a major improvement during the follow-up period.

Clinical characterization of patients who exhibited poor functional recovery throughout the follow-up period and were classified to cluster 4 in comparison to other final cohort participants is presented in Table 3. Being significantly younger, they had lower prevalence of main comorbidities and lower BMI. Cluster 4 patients required oxygen support marginally less frequently, but at the same time exhibited more extensive pulmonary involvement as assessed by the chest CT. Their laboratory parameters profile was similar to patients of clusters 1–3, with an exception of higher values of ESR and eGFR (the latter being explained by younger age and lesser comorbidity burden). There was no difference revealed in the smoking status or applied treatments.

**Table 3:** Clinical characteristics of patients with poor vs. satisfactory/good dynamics of functional recovery after hospitalization for COVID-19 as assessed by 6MWT.

Parameters	Cluster 4	Clusters 1 + 2 + 3	Difference	2-sided *p*
	(Poor recovery)	(Satisfactory recovery)	(95% CI)	
Subjects	27	72		
Female sex	10 (37)	34 (47)		0.364
Age, years	38.2 ± 11.0	54.9 ± 12.1	16.8 (11.4–22.1)	<0.001
Height, cm	173.8 ± 11.2	171.1 ± 9.0	−2.7 (−7.1 to 1.6)	0.216
Weight, kg	84.1 ± 16.3	87.7 ± 15.2	3.5 (−3.5 to 10.6)	0.324
BMI, kg/m^2^	27.8 ± 4.7	29.9 ± 4.2	2.1 (0.1–4.0)	0.038
*Comorbidities*				
Hypertension	6 (22)	34 (47)		0.024
Obesity	7 (26)	35 (49)		0.042
Diabetes mellitus, type 2	2 (7)	12 (17)		0.239
Charlson comorbidity index	0.2 ± 0.8	0.6 ± 0.9	0.4 (0.0–0.8)	0.054
Active smoking status	7 (26)	17 (24)		0.810
Intensive care unit admission	2 (7)	4 (6)		0.731
Pulmonary tissue involvement by CT, %	43.7 ± 8.8	29.6 ± 19.4	−14.1 (−26.7 to −1.5)	0.029
Minimal SpO_2_ during hospitalization, %	90.1 ± 5.5	87.9 ± 8.7	−2.2 (−5.8 to 1.4)	0.227
*Oxygen supplementation*				
Via nasal cannula	11 (41)	46 (64)		0.038
Noninvasive/invasive ventilation	2 (7)	4 (6)		0.731
*Laboratory parameters*				
Peak IL-6, pg/mL	10.4 [6.3–17.9]	15.4 [3.1–50.0]		0.550
Peak CRP, mg/L	30 [12–130]	24 [6–60]		0.238
Peak ESR, mm/h	36.5 ± 9.7	25.6 ± 12.8	−10.8 (−16.9 to −4.7)	<0.001
Peak procalcitonin, ng/mL	0.06 [0.05–0.08]	0.06 [0.04–0.09]		0.649
Peak D-dimer, ng/mL	223 [153–414]	219 [156–628]		0.623
Peak creatinine, μmol/L	99.5 ± 16.7	99.4 ± 17.5	−0.1 (−11.5 to 11.3)	0.986
Lowest eGFR, ml/min/1.73m^2^	82.9 ± 21.8	69.1 ± 17.5	−13.7 (−25.9 to −1.6)	0.027
*Treatment*				
Remdesivir	9 (33)	33 (46)		0.262
Dexamethasone	25 (93)	58 (81)		0.147
Methylprednisolone pulse therapy	17 (61)	43 (60)		0.769
Antibiotics	23 (85)	60 (83)		0.824

CI, confidence interval; BMI, body mass index; CT, computed tomography; IL-6, interleukin 6; CRP, C-reactive protein; ESR, erythrocyte sedimentation rate; eGFR, estimated glomerular filtration rate by CKD-EPI equation.

Despite their younger age, patients of cluster 4 at both visits showed 6MWD values close to those in clusters 1–3, resulting in significantly lower percent of predicted distance that was reached during the test (see Table 4). The mean 6MWD% increment between visits was more than two times less compared to the rest of study cohort. Oxygen saturation dynamics throughout the test did not explain the observed differences—patients of clusters 1–3 exhibited slightly and uniformly lower SpO_2_ values at both visits (which was expected given the older age and higher prevalence of obesity), with the differences to cluster 4 getting less pronounced at 1 month, when the observed values in both groups were getting closer to 99%. Baseline heart rate at visit 2 was higher in cluster 4 patients despite younger age and lesser burden of comorbidities. There were no significant differences revealed in values of the maximal reached heart rate, but patients of clusters 1–3 tended to slightly better sustain it throughout the test, resulting in higher percent of individually predicted maximum HR at 6 min.

**Table 4:** Six min walk test parameters in patients with poor vs. satisfactory/good dynamics of spontaneous functional recovery after hospitalization for COVID-19.

Parameters	Cluster 4	Clusters 1 + 2 + 3	Difference	2-sided *p*
	(Poor recovery)	(Satisfactory recovery)	(95% CI)	
*Distance*				
6MWD at visit 1, m	401 ± 48	390 ± 74	−10 (−41 to 21)	0.510
6MWD% at visit 1, %	59.4 ± 6.0	71.0 ± 11.3	11.6 (6.9–16.2)	<0.001
6MWD at visit 2, m	446 ± 56	468 ± 62	22 (5–50)	0.112
6MWD% at visit 2, %	66.6 ± 7.0	86.1 ± 9.4	19.4 (15.5–23.5)	<0.001
Delta 6MWD between visits, m	45 ± 30	78 ± 44	32 (14–51)	<0.001
Delta 6MWD% between visits, %	7.2 ± 4.2	15.1 ± 7.9	7.9 (4.6–11.2)	<0.001
*Heart rate*				
HR_base_ at visit 1, min^−1^	85.4 ± 11.3	81.1 ± 13.1	−4.2 (−10.0 to 1.6)	0.151
HR_max_ at visit 1, min^−1^	107.2 ± 15.1	104.4 ± 14.3	−2.8 (−9.6 to 4.0)	0.422
HR_max_ % at visit 1, %	59.2 ± 7.6	61.3 ± 7.7	2.1 (−1.4 to 5.7)	0.239
HR_fin_ % at visit 1, %	56.5 ± 8.3	58.5 ± 7.0	2.0 (−1.5 to 5.6)	0.250
HR_base_ at visit 2, min^−1^	86.4 ± 17.0	79.8 ± 13.5	−6.6 (−13.2 to 0.0)	0.048
HR_max_ at visit 2, min^−1^	111.6 ± 13.9	109.2 ± 14.2	−2.4 (−8.8 to 4.0)	0.463
HR_max_ % at visit 2, %	61.7 ± 7.9	64.6 ± 7.9	3.0 (−0.6 to 6.5)	0.106
HR_fin_ % at visit 2, %	58.5 ± 7.3	62.7 ± 7.2	4.2 (0.6–7.8)	0.021
*Oxygen saturation*				
SpO_2 base_ at visit 1, %	97.8 ± 1.1	96.8 ± 2.2	−1.0 (−1.9 to 0.1)	0.037
SpO_2 min_ at visit 1, %	95.7 ± 2.7	93.8 ± 4.4	−1.9 (−3.7 to 0.1)	0.036
SpO_2 fin_ at visit 1, %	96.8 ± 2.2	95.0 ± 4.1	−1.8 (−3.4 to 0.1)	0.042
SpO_2 drop_ at visit 1, %	2.1 ± 2.1	3.1 ± 2.5	1.0 (−0.1 to 2.1)	0.081
SpO_2 base_ at visit 2, %	98.4 ± 0.5	97.9 ± 0.9	−0.4 (−0.8 to 0.1)	0.015
SpO_2 min_ at visit 2, %	96.6 ± 0.9	95.5 ± 2.7	−1.1 (−2.1 to 0.0)	0.044
SpO_2 fin_ at visit 2, %	97.6 ± 0.8	97.0 ± 1.5	−0.6 (−1.2 to 0.0)	0.054
SpO_2 drop_ at visit 2, %	1.8 ± 1.0	2.5 ± 2.5	0.7 (−0.3 to 1.7)	0.163

CI, confidence interval; 6MWD, 6 min walk distance; 6MWD%, reached % of predicted 6 min walk distance; HR_base_, baseline heart rate; HR_max_, maximal reached heart rate; HR_max_ %, reached percent of the individual maximum heart rate; HR_fin_ %, percent of the individual maximum heart rate at 6 min; SpO_2 base_, baseline oxygen saturation; SpO_2 min_, minimal oxygen saturation; SpO_2 fin_, oxygen saturation at 6 min; SpO_2_, peak oxygen desaturation.

### 3.3. Prediction of poor recovery

As a first step in building a tool that would predict poor functional recovery during the first month following hospitalization for COVID-19 as assessed by the 6MWT, logistic regression analysis was performed, where assigning to cluster 4 was considered a poor outcome. Among the clinical parameters that were included to baseline marginal analysis, age showed the highest predictive value, followed by ESR; other significant predictors included eGFR, percentage of pulmonary involvement by CT, need for oxygen supplementation during hospitalization, SpO_2_ and mMRC dyspnea score pre-discharge, and history of hypertension (see Supplementary Table S1). Among the resulting stable models, two with the highest values of summarizing statistics are presented below:


$$\begin{array}{c} A.\mathrm{Logit}\;\left(p\right)=-9.61341-0.1833\times \mathrm{Age}+0.26644\times \mathrm{ESR}\\ +0.08738\times \mathrm{eGFR} \end{array}$$

where age, age (years); ESR, erythrocyte sedimentation rate (mm/h); eGFR, estimated glomerular filtration rate (CKD-EPI equation). (Somers’ D = 0.900, Kolmogorov–Smirnov statistic = 0.833, AUC in ROC analysis = 0.950).


$$\begin{array}{c} B.\mathrm{Logit}\;\left(p\right)=-497.662+0.683\times \mathrm{Height}+0.748\times \mathrm{CT}\%\\ +3.781\times {\mathrm{SpO}}_{2}-10,331\times \mathrm{Dyspnea} \end{array}$$

where height, height (cm); CT%, percentage of pulmonary involvement by CT during acute phase; SpO_2_, resting oxygen saturation on room air pre-discharge; dyspnea, mMRC dyspnea score pre-discharge. (Somers’ D = 0.952, Kolmogorov–Smirnov statistic = 0.92, AUC in ROC analysis = 0.976).

Model A included parameters easily obtainable during the first days of hospitalization, and Model B further improved the prediction quality based on anthropometrical features and parameters directly and indirectly assessing pulmonary involvement and its functional consequences. The detailed data on both models’ parameters estimates is presented in Supplementary Table S2.

**Figure 4:**
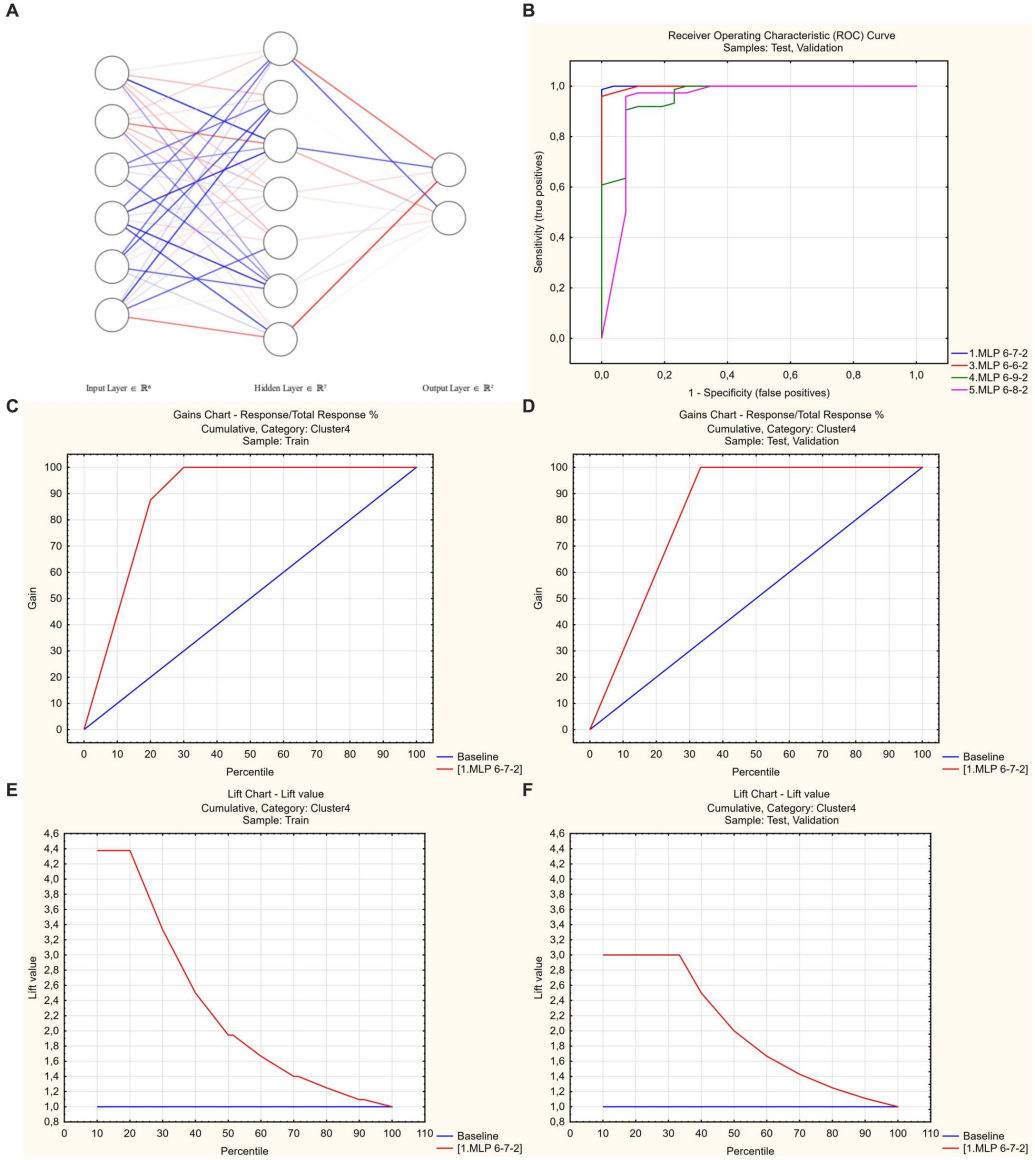
Artificial neural network to predict poor functional recovery after hospitalization for COVID-19. **(A)** 6-7-2 architecture. Link opacity and color are proportional to weight (red = positive, blue = negative). **(B)** Receiver operator characteristic analysis. Optimal model code = 1 (shown in blue), AUROC = 0.999. **(C,D)** Gains charts—train and test/validation samples. **(E,F)** Lift charts—train and test/validation samples.

As a final step in building a predictive tool, the unsupervised ML approach with the automated network search was used. All significant predictors that were detected earlier in logistic regression marginal analysis were included as initial input variables. Stepwise deletion of excessive inputs was subsequently performed, allowing to create an artificial neural network that only used simple clinical parameters easily assessible in all patients regardless of the clinical severity of disease—age, history of hypertension, requirement of oxygen supplementation during hospitalization, and ESR. The obtained network had 6-7-2 SANN architecture and was characterized by 100% predictive performance in the randomly selected test and validation subsets of study cases and 94% performance in cross-validation (Figure 4; source file available in open access at https://doi.org/10.5281/zenodo.7861928; see Supplementary material for information on network weights and connections, and instructions for use).

## 4. Discussion

Persistence of symptoms beyond the acute phase of COVID-19 has become a growing problem due to its effects on quality of life, physical performance, and cognitive function (1, 2). The observed wide spectrum of post-acute clinical manifestations has created the need for universalization of the set of outcomes to be used in long COVID related research which had been recently fulfilled in an international Delphi consensus study (26). The resulting core outcome list included, among others, fatigue, post-exertion symptoms and altered physical functioning, which are being referred to as most frequently imposing debilitating effect (8, 27).

Cardiopulmonary exercise test (CPET) remains the gold standard for the quantitative assessment of physical performance, given its high sensitivity and discriminatory ability in differentiation of exercise intolerance causes (28). At the same time, a high flow of newly-recovered post-COVID-19 patients calls for the use of simpler outcome measures with higher availability. Among the tests that fit this description, sit-to-stand (STS) and 6 min walk test (6MWT) are the most obvious choices due to the ease of use and being low-tech. Out of the two, 6MWT has some additional benefits that include better standardization (21), wider spectrum of obtained parameters, and more robust evidence of its diagnostic and prognostic significance both in the setting of chronic pulmonary conditions (17, 29) and post-acute SARS-CoV-2 infection (19). A recent meta-analysis has also confirmed the sensitivity of 6MWT in assessing the efficacy of post-COVID-19 pulmonary rehabilitation (30). In addition, 6MWT requires a sustained submaximal effort for a longer period of time compared to what is used in the STS test (30 to 60 s) and thus could increase the chance to unmask possible subclinical cardiopulmonary functional alterations.

This study reports on the results of comprehensive 6MWT in hospitalized COVID-19 patients that was performed 1–2 days pre-discharge at a baseline and after 1 month of spontaneous recovery and assessed the distance walked, changes of heart rate and SpO_2_ throughout the test.

The main findings at the baseline evaluation included a significant reduction in 6MWD (396 ± 71 m or 68.7 ± 12.4% compared to individually predicted values) that was associated with a 2.9 ± 2.5% maximal oxygen desaturation throughout the test and a modest 24.9 ± 17.5 bpm increment of heart rate that topped out at 61.1 ± 8.1% of individually predicted maximum. Re-evaluation at 1 month has revealed an improvement in most parameters, but the mean values of 6MWD% (81.8 ± 13.6), HR_max_ % (64.3 ± 8.2), and HR increment during the test (28.2 ± 12.0 bpm) remained suboptimal, thus objectively confirming the persistently impaired physical performance in study participants.

A large number of regression equations exists to predict individually expected 6MWD (17), and the selection of model naturally affects the interpretation of test data. Pursuing the reproducibility and comparability of obtained results, we have chosen to use classic equations by Enright et al. (23) that were derived from the cohort of patients aged 40 to 80 years using a 30 m hallway. Thus, systematic overestimation of predicted 6MWD in younger patients and shorter track effects had to be excluded as a possible source of bias in our study. To solve this problem, were-calculated the analyzed parameters using equations obtained at a 20 m walkway in 20–80 years old population (31) and those using log-linear regression to account for possible non-linear effects (32). None of alternatives used led to essential changes in the observed results, which, together with almost perfect linearity of the age—factual 6MWD scatterplots in the cohort used by Enright et al. (23) allows us to consider this factor irrelevant.

A meta-analysis by Ahmed et al. (30) highlights the beneficial effects of various modes of cardiopulmonary rehabilitation on the physical performance and measures of pulmonary function in both acute and chronic COVID-19. Importantly, the effect size of rehabilitation programs was much larger in initiation of intervention earlier compared to the studies where the program started at 35 or 70 days post discharge—the mean 6MWD difference between the baseline and follow-up was 83 (56–109) vs. 44 (20–68) m, *p* = 0.03. Regardless of the timing of baseline evaluation that ranged from the first week from the diagnosis to 70 days post-discharge, none of the 7 studies that reported the changes in the 6MWD have demonstrated a significant improvement in the control group that refrained from physical activity. The results of our observational study suggest that the real-life scenario of a post-discharge return to the usual routine imposes a significant training effect that might be comparable to that of rehabilitation program in a large proportion of COVID-19 convalescents, reaching as high as 78 ± 44 m in the cohort of patients with satisfactory post-discharge recovery.

As a tool to identify the patients with poor 1 month dynamics of spontaneous physical recovery as assessed by the 6MWT (and who, therefore, would hypothetically benefit the most from rehabilitation intervention), we applied the automatic clusterization of patients by the baseline values and between-visits increment of 6MWD%. This approach allowed to exclude the patients who did not have impressive improvement as a result of high base effect (cluster 3), and also those in whom suboptimal visit 2 parameters were resulting from significant baseline impairment but who were improving fast during follow-up (cluster 1, see Figure 3).

Compared to the remaining part of the study cohort, patients with poor “low base—low increment” trajectory of post-discharge recovery appeared to be younger (and hence leaner and less frequently hypertensive) but had higher ESR and more extensive pulmonary involvement as assessed by CT. The same parameters along with eGFR, need for oxygen supplementation, SpO_2_ and mMRC dyspnea score pre-discharge were predictors of attribution to the poor recovery cluster in the multivariate regression analysis that allowed to build two high quality logistic models based on early in-hospital (Model A, AUROC = 0.95) and pre-discharge parameters (Model B, AUROC = 0.98). One has to mention that the available cohort size was less compared to recommended minimum for logistic regression (33), hence no further downsizing it due to sampling in order to allow for subsequent internal validation was used.

With a purpose of further optimization of patients’ classification, unsupervised ML-based predictive model utilizing 6-7-2 SANN architecture was created that used age, history of hypertension, need for oxygen supplementation, and ESR as inputs, and had 100% performance in the random 30% test/validation subset of the study cohort and 94% predictive performance in cross-validation. Sample size assessment in ML projects is a problem with no consensus reached to date (34); in our study, the ratio of training cases to predictors number was 17.5:1, which was in line with the usual requirements used for ML projects (35). In addition, we applied a post-hoc assessment approach that has the benefit of being better fit to specific task, thus preventing the overestimation of minimally required sample size (25, 34). The combination of large input dataset effect size (Cohen’s *d* = 0.739), high accuracy of the acquired model and its reproducibility in cross-validation attested to its validity and absence of significant overfitting and random effects (25).

The long-lasting decrease of physical performance is one of the typical features of post-COVID syndrome, as has been shown in a number of studies in the form of decreased oxygen uptake during CPET (28). Being an integrative parameter, the latter might have heterogenous underlying mechanisms in different phenotypes of long COVID.

There is evidence supporting the role of decreased diffusing lung capacity (DLCO) and, to a lesser extent, restrictive pulmonary physiology (36, 37) in this phenomenon. The study by Orzes et al. (38) reveals a long-term preservation of functional pulmonary abnormalities that were present in 53% of patients at 3 months and in 38% at 6 months after discharge, with the combination of decreased DLCO and restrictive changes being the dominant pattern. The reported rates of respiratory abnormalities were also in line with the previous studies that revealed 53 to 71% prevalence of subnormal DLCO and 12% to 21% of restrictive changes at the term of 4 to 6 weeks (39, 40). These findings are supported by the meta-analysis by Guo et al. (36) that underlines the poor dynamics of spontaneous reversal of residual pulmonary function alterations starting from the term of 1 month post-discharge and extending beyond 6 months, thus supporting the concept of early identification of patients at risk with a subsequent focus on their targeted rehabilitation.

An additive burden on oxygen delivery system may also be imposed by co-existing mild impairment in cardiac function manifested as lower stroke volume, global longitudinal strain and high incidence of diastolic dysfunction (41, 42). At the same time, the grade of detected alterations in the mentioned central Wasserman gears (43) does not completely account for the decrease of oxygen uptake that is being detected in post-acute COVID-19 patients.

Dysautonomia and chronotropic incompetence have also been shown to contribute to the observed decrease in oxygen uptake (44, 45). This concept is being corroborated by the results of our study, where patients with poor post-discharge functional recovery, despite their younger age, exhibited higher baseline heart rate and the tendency to lower percentage of individual maximum heart rate that was reached during the test. Finally, peripheral mitochondrial function might also be altered in post-COVID syndrome further contributing to decreased oxygen utilization (28).

Given the heterogeneity of possible mechanisms of reduction of functional capacity, pathophysiological phenotyping of long COVID population might prove to be beneficial in the context of targeted treatment strategies. At the same time, their proper development and implementation is a lengthy process with unclear timeline. Cardiopulmonary rehabilitation remains the only intervention that has been shown to date to improve physical performance in patients with long COVID syndrome (14, 15), and there is no reason to believe that newly developed treatments might cause a decrease in its use. With a continuously growing post-COVID population, rehabilitation system capacity gets at times overwhelmed, calling for optimization of candidate selection for the supervised training programs which would allow for more effective use of available resources.

A lot of studies have been devoted to exploring the spectrum of long COVID symptoms and identifying patients at risk of its development (37). At the same time, majority of them were using self-reported outcome measures of physical performance that are susceptible to excessive variability related to differences in individual perception. Among the predictors of objectively measured physical recovery outcomes in the post-acute COVID-19 setting, we have identified mentioning of PaO_2_/FiO_2_ during hospitalization and extensity of radiological abnormalities (9), frailty and length of hospital stay (46), persistence of fatigue and dyspnea (47), which was in line with the map of predictors detected in our cohort.

To our knowledge, this is the first study proposing a set of predictive tools for the assessment of expected physical recovery dynamics by the 6MWT in the early post-discharge period after COVID-19 basing on sets of inputs optimized for their easy and early availability during in-hospital period (similar approach has been described recently for the prediction of radiological recovery (48)). The proposed ML-based classification model may be used as a self-sufficient tool after external validation on the local cohort of hospitalized COVID-19 patients, or as a concept for development of similar models that would account for features of local population and currently prevailing SARS-CoV-2 variants.

### 4.1. Limitations

The results of our study could be susceptible to center-related confounding effects, with frequent use of methylprednisolone pulse therapy being the most notable difference compared to the commonly applied standards. However, no correlations were found between its use and parameters of 6MWT at both visits (with an exception of weak negative correlations to SpO_2_ values pre-discharge which seemed to be residual representation of stronger association with low SpO_2_ level on admission). Patients with more severe course of COVID-19 could be more likely to remain oxygen-dependent pre-discharge and thus unable, or be unwilling to participate, and those with severe concomitant pathology were not included to the study to omit possible confounding effects, presenting the source of selection bias. At the same time, the prevalence of main comorbidities in the study cohort was in line with previously published reports, and thus the general picture was unlikely to be altered due to the latter factor. Finally, the spectrum of currently prevailing SARS-CoV-2 variants has changed compared to the time of enrollment to our study, and a higher proportion of patients has since received vaccination. Thus, caution should be used in generalizing the results of this study to the current practice of long COVID care.

## 5. Conclusion

COVID-19 survivors were characterized by decreased physical performance pre-discharge as assessed by the 6MWT and did not completely restore their functional status after 1 month of spontaneous recovery, with signs of altered blood oxygenation and dysautonomia contributing to the observed changes. Patients with poor “low base—low increment” trajectory of post-discharge recovery were characterized by younger age but more extensive pulmonary involvement and higher peak ESR values. Poor post-discharge recovery in the study cohort was predictable by the means of SANN-based machine learning classification model that used age, history of hypertension, need for oxygen supplementation, and ESR as inputs.

## Data availability statement

The raw data supporting the conclusions of this article will be made available by the authors, without undue reservation.

## Ethics statement

The studies involving human participants were reviewed and approved by Ethical committee of Kharkiv National Medical University. The patients/participants provided their written informed consent to participate in this study.

## Author contributions

OH: study design, literature search, data collection, data analysis, data interpretation, writing manuscript, and revision of the manuscript. TA: study design, data interpretation, and revision of the manuscript. All authors contributed to the article and approved the submitted version.

## Acknowledgments

The authors would like to thank M. Yerzina for the technical support in data collection and to acknowledge the work of the hospital staff: A. Bobeyko, V. Byzov, V. Blazhko, E. Khodosh, N. Matyash, O. Morozova, L. Kolisnyk, I. Talalay, V. Kozlov, and L. Avdeyeva, who have set conditions for the proper functioning of the clinic.

## Conflict of interest

The authors declare that the research was conducted in the absence of any commercial or financial relationships that could be construed as a potential conflict of interest.

## Publisher’s note

All claims expressed in this article are solely those of the authors and do not necessarily represent those of their affiliated organizations, or those of the publisher, the editors and the reviewers. Any product that may be evaluated in this article, or claim that may be made by its manufacturer, is not guaranteed or endorsed by the publisher.

## Supplementary material

The Supplementary material for this article can be found online at: https://www.frontiersin.org/articles/10.3389/fmed.2023.1212678/full#supplementary-material
