# Supplementary material for: Spontaneous physical functional recovery after hospitalization for COVID-19: insights from a 1 month follow-up and a model to predict poor trajectory
Source: Front Med (Lausanne). 2023 Jul 20;10:1212678. doi: 10.3389/fmed.2023.1212678 (PMC10399450; doi:10.3389/fmed.2023.1212678)
Supplement: Supplementary file 1 [file Data_Sheet_1.PDF]

Supplementary Material

Spontaneous Physical Functional Recovery After Hospitalization for COVID-19: Insights From a 1-Month Follow-Up and a Model to Predict Poor Trajectory

Oleksii Honchar*, Tetyana Ashcheulova

*** Correspondence:**
Corresponding Author
ov.honchar@knmu.edu.ua

2 Material and Methods

Study exclusion criteria:

- stage D chronic heart failure;
- acute heart failure;
- myocardial infarction;
- permanent atrial fibrillation;
- stroke within 6 months;
- severe uncontrolled hypertension;
- significant valvular heart disease;
- active cancer or systemic autoimmune pathology;
- inability to provide an informed consent;
- persisting O2 supplementation dependence by the time of discharge.

3 Results

The source file of the final machine learning classification model is available in open access at https://doi.org/10.5281/zenodo.7861928 and requires an input dataset with the following variables:

- Age = age (years);
- ESR = erythrocyte sedimentation rate (mm/h);
- Tx-O2 = oxygen supplementation during treatment (2=Yes, 1=No);
- HT = history of hypertension (2=Yes, 1=No).

The output codes for predicted recovery pattern include “Good” for good/satisfactory recovery, “Bad” for poor recovery.

Supplementary Tables

Supplementary table 1. Baseline marginal analysis of potential predictors in multivariate logistic regression analysis (poor outcome = Cluster 4 assignment).

Effect	Cluster 4 vs 1,2,3 – Marginal table					
	Level	Somers' D	Estimate	Pr>Chi.Sqr	df	Sample
Age		0,704782	-0,107234652	0,00000247492865	1	Full
Waist circumference		0,149688	-0,0292103607	0,0853573804	1	Full
Peak C-reactive protein		0,175926	0,000921898612	0,788719766	1	Full
Peak White blood cells count		0,043636	0,0849799102	0,143060949	1	Full
Peak ESR		0,447552	0,0747975206	0,00303868612	1	Full
Peak Ferritin		-0,09091	-0,00008890024	0,679309845	1	Full
Lowest eGFR		0,348485	0,0367623121	0,044075721	1	Full
Systolic blood pressure		0,010684	-0,00159894634	0,909772287	1	Full
Diastolic blood pressure		0,141026	-0,0282288529	0,176734392	1	Full
Resting heart rate pre-discharge		0,179487	0,0249856795	0,180390353	1	Full
Resting SpO2 pre-discharge		0,203742	0,29879043	0,0489672496	1	Full
mMRC dyspnea pre-discharge		0,138889	-0,325615941	0,138359105	1	Full
Peak Creatinine		0,075758	0,00359983578	0,849417689	1	Full
Peak Interleukin-6		-0,109375	0,00396022491	0,611705618	1	Full
Peak D-dimer		0,083333	-0,000602898653	0,44475872	1	Full
Peak Procalcitonin		0,04902	3,6851939	0,172308055	1	Full
Hemoglobin		0,171329	-0,0250136755	0,168813312	1	Full
Height		0,170478	0,0308821361	0,204039442	1	Full
Weight		0,087318	-0,0105853243	0,468715068	1	Full
Body mass index		0,209979	-0,0873889421	0,0905488358	1	Full
Pulmonary affection by CT		0,504	0,0441515048	0,032392768	1	Full
Minimal Sp02 during disease		0,093555	0,0426158165	0,208031768	1	Full
Sex	1	0,074844	0,15374235	0,509119214	1	Full
Tx: Dexamethasone	1	0,112266	-0,514809708	0,194478555	1	Full
Tx: Remdesivir	1	0,137255	0,287682072	0,246536219	1	Full
Tx: O2 supplementation	1	0,237006	0,483220258	0,0393339338	1	Full
Active smoking	1	0,012474	0,0344964357	0,898070613	1	Full
History of hypertension	1	0,22869	0,520726937	0,0454691598	1	Full

Note. * Assessment was performed using the methodology for the simplified RALE score as proposed by Wong et al. [1], mean value of the reported % range was taken for analysis. ESR – erythrocyte sedimentation rate, eGFR – estimated glomerular filtration rate by CKD-EPI equation, CT – computed tomography, SpO2 – capillary blood oxygen saturation, Tx – treatment.

Supplementary table 2. Parameters of logistic regression models predicting poor functional recovery after hospitalization for COVID-19.

Effect	Estimate	Standard Error	Wald Stat.	Lower CL 95,0%	Upper CL 95,0%	p
Model A (Somers' D = 0,900, Kolmogorov-Smirnov statistic = 0,833, AUC in ROC analysis = 0,95)						
Intercept	-9,61341	5,491331	3,064779	-20,3762	1,149406	0,080006
Age, years	-0,18330	0,075803	5,847117	-0,3319	-0,034727	0,015603
ESR, mm	0,26644	0,107368	6,157969	0,0560	0,476875	0,013082
eGFR, ml/min/1,73m^2	0,08738	0,043787	3,982709	0,0016	0,173206	0,045970
Model B (Somers' D = 0,952, Kolmogorov-Smirnov statistic = 0,92, AUC in ROC analysis = 0,976)						
Intercept	-497,662	218,7272	5,176822	-926,359	-68,9645	0,022890
Pulmonary affection by CT, % *	0,748	0,3412	4,811930	0,080	1,4171	0,028263
mMRC dyspnea pre-discharge	-10,331	4,8590	4,520904	-19,855	-0,8079	0,033483
Resting SpO2 pre-discharge	3,781	1,6803	5,062599	0,487	7,0739	0,024448
Height, cm	0,683	0,3166	4,648706	0,062	1,3032	0,031077

Note. * Assessment was performed using the methodology for the simplified RALE score as proposed by Wong et al. [1], mean value of the reported % range was taken for analysis. ESR – erythrocyte sedimentation rate, eGFR – estimated glomerular filtration rate by CKD-EPI equation, CT – computed tomography, SpO2 – capillary blood oxygen saturation.

Supplementary table 3. Connections and weight values of the final machine learning model predicting poor functional recovery after hospitalization for COVID-19.

Weight ID	Connections<br>1.MLP 6-7-2	Weight values<br>1.MLP 6-7-2
1	ESR --> hidden neuron 1	-2,7259
2	Age --> hidden neuron 1	4,2872
3	Tx-O2(1) --> hidden neuron 1	0,8381
4	Tx-O2(2) --> hidden neuron 1	0,7631
5	HT(1) --> hidden neuron 1	-0,4200
6	HT(2) --> hidden neuron 1	1,9568
7	ESR --> hidden neuron 2	-10,5107
8	Age --> hidden neuron 2	4,3939
9	Tx-O2(1) --> hidden neuron 2	-1,1254
10	Tx-O2(2) --> hidden neuron 2	6,3767
11	HT(1) --> hidden neuron 2	6,3525
12	HT(2) --> hidden neuron 2	-0,9997
13	ESR --> hidden neuron 3	-16,5367
14	Age --> hidden neuron 3	-3,5896
15	Tx-O2(1) --> hidden neuron 3	5,5946
16	Tx-O2(2) --> hidden neuron 3	-0,9524
17	HT(1) --> hidden neuron 3	-3,3104
18	HT(2) --> hidden neuron 3	7,9791
19	ESR --> hidden neuron 4	-2,5585
20	Age --> hidden neuron 4	3,2819
21	Tx-O2(1) --> hidden neuron 4	-0,8295
22	Tx-O2(2) --> hidden neuron 4	3,2925
23	HT(1) --> hidden neuron 4	0,2025
24	HT(2) --> hidden neuron 4	2,2377
25	ESR --> hidden neuron 5	-0,3446
26	Age --> hidden neuron 5	2,4628
27	Tx-O2(1) --> hidden neuron 5	-2,7411
28	Tx-O2(2) --> hidden neuron 5	3,0526
29	HT(1) --> hidden neuron 5	-1,3446
30	HT(2) --> hidden neuron 5	1,5986
31	ESR --> hidden neuron 6	-4,5837
32	Age --> hidden neuron 6	-2,9924
33	Tx-O2(1) --> hidden neuron 6	2,0616
34	Tx-O2(2) --> hidden neuron 6	-2,6788
35	HT(1) --> hidden neuron 6	-0,9331

36	HT(2) --> hidden neuron 6	0,4204
37	ESR --> hidden neuron 7	2,7358
38	Age --> hidden neuron 7	-13,6078
39	Tx-O2(1) --> hidden neuron 7	5,1163
40	Tx-O2(2) --> hidden neuron 7	-2,6670
41	HT(1) --> hidden neuron 7	-0,0350
42	HT(2) --> hidden neuron 7	2,3624
43	input bias --> hidden neuron 1	1,5951
44	input bias --> hidden neuron 2	5,2955
45	input bias --> hidden neuron 3	4,6569
46	input bias --> hidden neuron 4	2,4770
47	input bias --> hidden neuron 5	0,2995
48	input bias --> hidden neuron 6	-0,5301
49	input bias --> hidden neuron 7	2,3429
50	hidden neuron 1 --> Outcome(Bad)	-2,5853
51	hidden neuron 2 --> Outcome(Bad)	-3,5589
52	hidden neuron 3 --> Outcome(Bad)	-4,6391
53	hidden neuron 4 --> Outcome(Bad)	-1,1919
54	hidden neuron 5 --> Outcome(Bad)	2,2517
55	hidden neuron 6 --> Outcome(Bad)	-3,0447
56	hidden neuron 7 --> Outcome(Bad)	2,9635
57	hidden neuron 1 --> Outcome(Good)	2,6671
58	hidden neuron 2 --> Outcome(Good)	3,5528
59	hidden neuron 3 --> Outcome(Good)	4,6529
60	hidden neuron 4 --> Outcome(Good)	1,2422
61	hidden neuron 5 --> Outcome(Good)	-2,2630
62	hidden neuron 6 --> Outcome(Good)	2,9472
63	hidden neuron 7 --> Outcome(Good)	-2,8994
64	hidden bias --> Outcome(Bad)	-0,7167
65	hidden bias --> Outcome(Good)	0,6011

Note. Training algorithm = BFGS 18; Error function = Entropy; Hidden activation = Tanh; Output activation = Softmax.
